# 腰果酚键合硅胶固定相的制备及其色谱性能

**DOI:** 10.3724/SP.J.1123.2021.12023

**Published:** 2022-06-08

**Authors:** Lei ZENG, Lijuan JIANG, Xingdong YAO, Ting WANG, Bo’an SHI, Fuhou LEI

**Affiliations:** 1.广西民族大学化学化工学院, 广西林产化学与工程重点实验室, 林产化学与工程国家民委重点实验室, 广西林产化学与工程协同创新中心, 广西 南宁 530006; 1. School of Chemistry and Chemical Engineering, Guangxi University for Nationalities, Guangxi Key Laboratory of Chemistry and Engineering of Forest Products, Key Laboratory of Chemistry and Engineering of Forest Products of State Ethnic Affairs Commission, Guangxi Collaborative Innovation Center for Chemistry and Engineering of Forest Products, Nanning 530006, China; 2.湖北民族大学化学与环境工程学院, 湖北 恩施 445000; 2. School of Chemical and Environmental Engineering, Hubei Minzu University, Enshi 445000, China

**Keywords:** 腰果酚, 键合固定相, 色谱性能, 中药分离, cardanol, bonded stationary phase, chromatographic properties, separation of traditional Chinese medicines

## Abstract

天然产物作为一种绿色低毒、来源广泛、功能位点丰富的单体,已被广泛应用于色谱固定相的研制与开发。该文以天然可再生资源腰果酚为配体,通过一步法开环反应将其接枝到由*γ*-缩水甘油醚氧丙基三甲氧基硅烷(KH-560)修饰的硅胶上,制备得到腰果酚键合硅胶固定相。利用傅里叶红外光谱、元素分析、热失重分析和N_2_吸附脱附实验对固定相进行表征,结果表明成功制备了腰果酚键合硅胶色谱固定相。采用Tanaka实验试剂、烷基苯、多环芳香烃、苯酚类化合物和芳香族位置异构体为探针评价其分离性能和保留机制,并与C_18_柱进行对比。研究发现,腰果酚键合固定相除疏水作用外,还具有*π-π*和氢键作用。基于上述保留作用,腰果酚键合硅胶固定相对测试探针表现出良好的分离性能。重复进样10次,各探针保留时间的RSD为0.052%~0.079%,峰面积的RSD为0.104%~0.847%,峰高的RSD为0.081%~0.272%,表明该色谱柱具有良好的重复性和稳定性。此外,腰果酚键合硅胶色谱柱对中药喜树果和吴茱萸果的粗提物具有良好的分离性能,验证了其在实际样品分析中的巨大潜力。将天然产物腰果酚用于色谱固定相的制备,为分离纯化喜树碱和吴茱萸提供了新的方法,同时拓展了腰果酚在色谱分离材料方面的应用。

## Abstract (en)

As green, less toxic, widely available, and site-rich functional ligands, natural products are widely used for the development of chromatographic stationary phases. In this work, a novel stationary phase, cardanol-bonded on silica (CBS) was prepared using *γ*-glycidoxypropyltrimethoxysilane (KH-560) as the coupling reagent and cardanol as the functional ligand. The synthesized stationary phase was characterized by Fourier transform-infrared spectra (FT-IR), thermogravimetric analysis (TGA), elemental analysis (EA), and N_2_ adsorption-desorption analysis. The results revealed that cardanol was successfully immobilized on the surface of spherical silica by the ring-opening reaction of the epoxy groups in phenolic hydroxyl. The retention mechanism and chromatographic performance of the CBS column were further evaluated and compared with those of a commercial C_18_ column using different classes of analytes, e. g., Tanaka standard test mixtures, alkylbenzenes, polycyclic aromatic hydrocarbons (PAHs), phenols, and aromatic positional isomers. The retention of alkylbenzenes under different chromatographic conditions revealed that the CBS column was a typical reversed-phase liquid chromatographic column, similar to the commercial C_18_ column. From the results of the Tanaka test, it was concluded that CBS could provide various interactions for different solutes e. g., hydrogen bonding and *π-π* interactions, along with hydrophobic interactions. The synergistic effects resulting from the aromatic rings, the hydroxyl and alkyl linkers in the new stationary phase improved the separation selectivity via multiple retention mechanisms. Based on these interactions, different solute probes such as hydrophobic alkylbenzenes, PAHs, and phenols were successfully separated in the reversed-phase liquid chromatography (RPLC) mode. For example, the aromatic positional isomers *o*-terthenyl, *m*-terphenyl, and triphenylene were used to investigate the chromatographic performance of the CBS column. These PHAs were baseline separated with good peak shapes. The resolution of *m*-terphenyl and triphenylene was as high as 6.81, while the two isomers could not be separated on the C_18_ column under the same chromatographic conditions. The repeatability and column stability of the CBS column was evaluated, and excellent repeatability and column stability were observed. The relative standard deviations (RSDs) of the retention time, peak area, and peak height for alkylbenzenes with 10 replicate injections were 0.052%-0.079%, 0.104%-0.847%, and 0.081%-0.272%, respectively. Traditional Chinese medicines have contributed notably to the Chinese civilization and human health. However, the complicated chemical compositions, unclear medicinal action mechanisms, and low purification efficiency for the traditional Chinese medicines have limited further development. Therefore it is necessary to establish an efficient, simple and feasible method for the separation and purification of herbal medicines. HPLC has been widely used in traditional Chinese medicines for the separation and detection of various components. In order to explore the CBS column for analysis of the traditional Chinese medicines, the ethanol extracts of fruits of *Evodiae fructus* and *Camptotheca acuminata* were used to test the separation performance of this column. The resolution of camptothecin from the preceding and following impurity peaks was 4.23 and 2.71. The resolution between evodiamine and rutaecarpin was 5.43, while the resolution from the adjacencies of impurity peaks was 2.20 and 1.69, respectively. The above mentioned results indicated that the CBS column shows good separation performance for the main active ingredients in the ethanolic extracts of these drugs, this validating its great potential for the analysis of real samples. Overall, the present study not only provides a new approach for the preparation of chromatographic stationary phases but also opens a new possibility for the separation and purification of camptothecin and evodiamine in real samples. This is an extension of the application of cardanol to chromatographic separation materials.

高效液相色谱因其分析效率高、选择性好、操作简单,已经被广泛应用于化学化工、食品检测、药物测试、环境监测等领域^[1⇓-3]^。随着分析和检测样品的多样化和复杂化,具有不同分离特性的色谱固定相应运而生并得到应用,其中化学键合固定相因其稳定性好、柱效高和重复性好等优点被广泛应用。硅胶由于具有机械强度高、比表面积大、孔结构丰富和易于修饰的优点,常作为键合固定相的基质。作为各种分离模式建立和发展的基础,新型色谱固定相的制备与应用已成为色谱研究工作的前沿和热点^[4⇓-6]^。

天然可再生资源作为一种绿色环保、来源广泛的原料已经被广泛应用于科学研究和工业化生产^[7]^,这对替代化石能源和实现“零碳排放”具有重要意义。天然化合物独特的化学结构和性能,使其在色谱固定相研制与开发方面也展现出极大的应用潜能^[8⇓-10]^。腰果酚作为一种从腰果壳油中提取的农业副产品,具有来源丰富、可再生和价格低廉等优点^[11,12]^。腰果酚是一种带有15个碳原子烷基链的间烷基苯酚混合物,其烷基链的不饱和度为0~3^[13,14]^。腰果酚兼具苯环的刚性、烷基链的柔性及酚羟基的反应活性,其独特的化学结构使其成为近年来生物基材料领域的研究热点之一,被广泛用于生物基树脂^[15,16]^、表面活性剂^[17]^和合成涂料^[18]^的制备。目前将腰果酚直接应用于色谱分离材料并对其分离性能进行评价的报道较少^[19]^。

本文以腰果酚为配体,*γ*-缩水甘油醚氧丙基三甲氧基硅烷(KH-560)为偶联剂,通过化学键合法制备得到腰果酚键合硅胶色谱固定相(CBS)。以烷基苯、多环芳烃、苯酚类物质和Tanaka实验试剂为探针,对其色谱性能进行评价,探讨其保留机制,并发掘该固定相在中药活性成分分离纯化研究中的应用价值。

## 1 实验部分

### 1.1 仪器与试剂

NicoletiS10傅里叶变换红外光谱仪(美国Nicolet公司); Z0050716装柱机(美国Scientific Systems公司);不锈钢色谱柱(250 mm×4.6 mm,大连依利特分析仪器有限公司); Mettler Toledo TGA/DSC3+同步热分析仪(瑞士梅特勒-托利多公司); ASAP 2020比表面积及氮气吸附仪(美国Micromeritics公司); HS3120超声波发生器(昆山市超声仪器有限公司); LC-20AD型高效液相色谱仪和SIL-20A自动进样器(日本岛津); C_18_色谱柱(250 mm×4.6 mm, 5 μm,日本岛津);超纯水机(Sartorius arium pro,德国)。

腰果酚(纯度98%,广西梧州日成林产化工有限公司);硅胶(5 μm,苏州纳微科技公司); *γ*-缩水甘油醚氧丙基三甲氧基硅烷(纯度98%,阿拉丁试剂公司);甲醇和乙腈(HPLC纯,赛默飞试剂公司);烷基苯、多环芳烃和苯酚衍生物均为分析纯(国药试剂公司);喜树碱、吴茱萸碱和吴茱萸次碱均为标准品(纯度98%,阿拉丁试剂公司);苯胺、尿嘧啶、咖啡因均为分析纯(麦克林试剂公司);吴茱萸果和喜树果干样(采自广西南宁)。

### 1.2 腰果酚键合硅胶色谱柱的制备

#### 1.2.1 腰果酚键合硅胶固定相的制备

腰果酚键合硅胶固定相的制备过程如图1所示,先用体积分数为30%的盐酸溶液对硅胶进行活化,在N_2_保护下80 ℃反应12 h,反应结束冷却至室温,用去离子水洗涤至中性后,再用无水乙醇洗涤。放入真空干燥箱80 ℃干燥12 h,制得活化硅胶。将15 g活化硅胶置于单口烧瓶中加入150 mL无水甲苯,搅拌条件下加入3 mL无水三乙胺及15 mL KH-560。在N_2_保护下110 ℃反应24 h,冷却至室温并依次用丙酮、甲醇、无水乙醇洗涤,最后真空干燥12 h,制备得到环氧硅胶。称取环氧硅胶5.0 g置于单口烧瓶中,加入50 mL无水甲苯,搅拌条件下加入1.0 g腰果酚和3滴高氯酸,在N_2_保护下90 ℃反应24 h,反应结束冷却至室温,依次用丙酮、无水乙醇和去离子水洗涤,并用无水乙醇进行索氏提取12 h,在80 ℃下真空干燥12 h,得到腰果酚键合硅胶固定相。

**图1:**
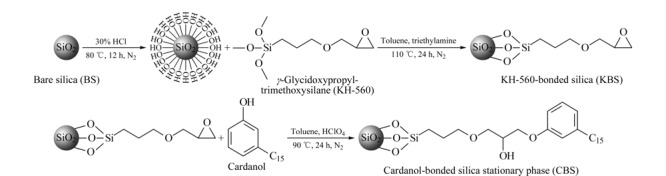
腰果酚键合硅胶色谱固定相的制备

#### 1.2.2 色谱柱的装填

采用匀浆法装柱,以异丙醇作为匀浆液,乙醇为顶替液。将4.5 g固定相加入到匀浆液中超声5 min使其混匀分散。在60 MPa的压力下将固定相装填到色谱柱中(250 mm×4.6 mm),保持压力25 min。装填完毕,以甲醇为流动相冲洗色谱柱,备用。

### 1.3 样品制备

#### 1.3.1 色谱测试标准品的制备

烷基苯、多环芳烃、咖啡因、苯酚、酚类化合物、苯胺、尿嘧啶均用甲醇超声溶解;喜树碱、吴茱萸碱和吴茱萸次碱均用乙腈超声溶解,过膜备用。

#### 1.3.2 吴茱萸粗提物与喜树果粗提物的制备

采用连续回流提取法制备喜树果粗提物^[10]^。将喜树果干燥,粉碎,过100目筛。称取50 g喜树果粉末,用纱布包好置于索氏提取器中,用75%(v/v)乙醇水溶液提取3次,每次1 h,合并3次粗提液,抽滤,除去喜树果滤渣。再用氯仿对醇提液进行萃取,收集氯仿层溶液并浓缩,制得喜树果浸膏。取喜树果浸膏5.0 mg,用乙腈定容至25 mL,用微孔滤膜过滤后得到喜树果粗提物溶液。吴茱萸粗提物采用同样方法制得。

## 2 结果与讨论

### 2.1 腰果酚键合硅胶固定相的表征

分别采用傅里叶红外光谱、元素分析、N_2_吸附脱附实验和热失重分析对CBS固定相进行表征。采用Vario EL cube元素分析仪分别对裸硅胶、环氧硅胶和腰果酚键合固定相中的C、H元素含量进行测定(见表1)。相较于裸硅胶而言,环氧硅胶中C和H元素的含量分别增加至8.465%和1.564%,表明成功制得环氧硅胶。腰果酚键合固定相的C和H元素的含量分别增至15.130%和2.181%,表明成功制得腰果酚键合硅胶固定相。

**表1:** 硅胶、环氧硅胶和固定相的元素分析结果

Analyte	Elemental contents/%	
	C	H
BS	0.735	1.121
KBS	8.465	1.564
CBS	15.130	2.181

通过N_2_吸附-脱附实验,对裸硅胶、环氧硅胶和固定相的BET比表面积和孔结构进行表征,结果如表2所示。随着对裸硅胶进一步的化学修饰,BET比表面积的数值逐渐下降,表明已成功地将硅烷偶联剂和腰果酚键合到硅胶上。此外,经修饰后环氧硅胶和固定相的孔体积和孔径均减小,说明在修饰过程中硅烷偶联剂和腰果酚不仅键合在硅胶表面,而且还进入硅胶的孔道中。

**表2:** BET比表面积和孔径数据

Analyte	BET surface area/ (m^2^/g)	Pore volume/ (cm^3^/g)	Pore size/ nm
BS	354.82	0.847	7.65
KBS	293.27	0.632	6.30
CBS	212.16	0.458	6.28

傅里叶红外光谱表征见图2。由图2a可以明显观察到裸硅胶在3454 cm^-1^处和972 cm^-1^处有明显的吸收峰,分别归属为Si-OH的伸缩振动峰和弯曲振动峰。由图2b可以明显地观察到,与裸硅胶相比,环氧硅胶在3454 cm^-1^处Si-OH的伸缩振动峰明显减弱,972 cm^-1^处Si-OH的弯曲振动峰明显消失,并在2941 cm^-1^和2864 cm^-1^处出现C-H的伸缩振动峰,表明成功将偶联剂KH-560修饰到裸硅胶上。由图2c可以观察到,2941和2864 cm^-1^处的吸收峰明显增强,在1626和1460 cm^-1^处出现芳环骨架C=C的伸缩振动峰;1093 cm^-1^处的Si-O和C-O重叠吸收峰变宽,表明成功将腰果酚键合到环氧硅胶上。

**图2:**
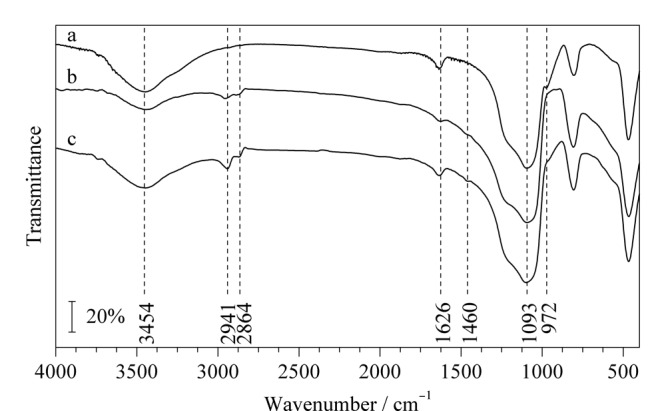
(a)硅胶、(b)环氧硅胶和(c)腰果酚键合硅胶固定相的红外光谱图

通过热失重分析评价CBS固定相的热稳定性,如图3所示。3种测试物在25~150 ℃处出现一个较小的失重现象,这是由于测试物脱水造成的。环氧硅胶在400 ℃处出现明显的失重现象,CBS固定相分别在330 ℃和415 ℃出现明显的失重现象,表明330 ℃处的失重现象是由于腰果酚热分解造成的。在800 ℃时,相较于裸硅胶而言,CBS固定相与环氧硅胶分别存在15.51%和10.48%的质量损失。上述结果表明,实验成功地将腰果酚键合到硅胶上,并且制备出的固定相具有良好的热稳定性。

**图3:**
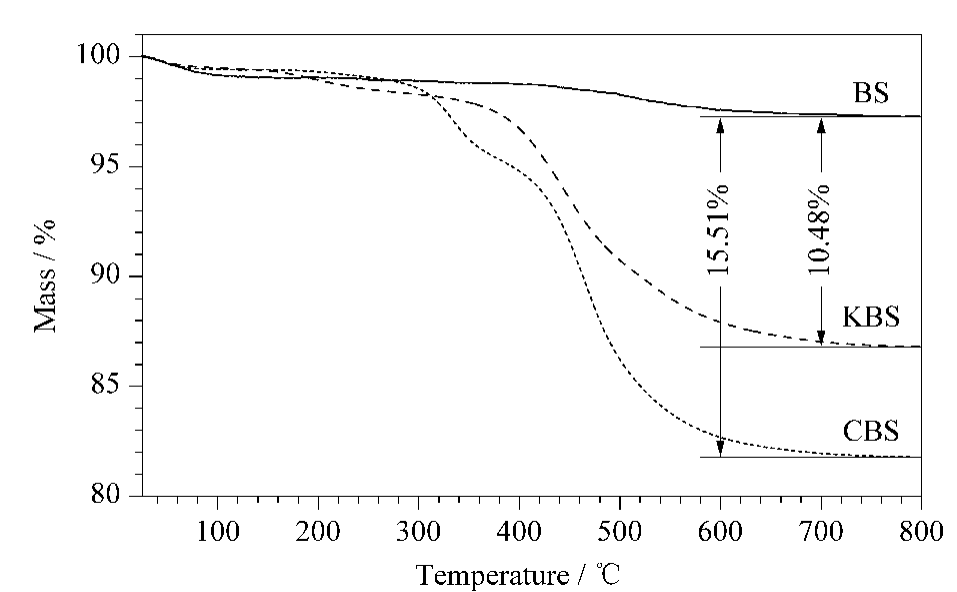
硅胶、环氧硅胶和腰果酚键合硅胶固定相的热失重曲线图

### 2.2 CBS柱的保留机制

以6种烷基苯为探针,考察CBS色谱柱的保留机制。各探针保留因子的对数(log *k*)与流动相中甲醇含量的变化关系如图4所示,随着流动相中甲醇比例从60%增加到85%,烷基苯在色谱柱上的保留能力呈现逐渐减弱的趋势,符合反相色谱保留特征,表明CBS柱具有反相色谱保留机制。

**图4:**
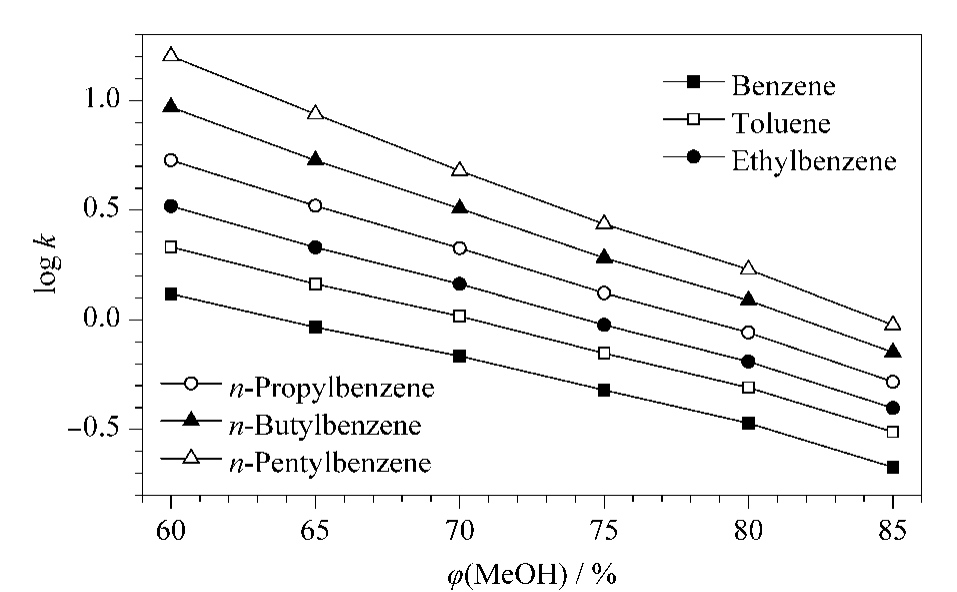
CBS柱上烷基苯保留因子的对数(log *k*)与甲醇体积分数的关系

基于CBS色谱柱的反相保留特征,为一步评价疏水作用在溶质保留中的作用,以烷基苯保留因子的对数log *k*与烷基苯所含烷基链长度进行线性拟合,评价其疏水性^[20]^,并与C_18_柱对比,结果如图5所示。在相同色谱条件下CBS柱和C_18_柱上的log *k*与其烷基链长度之间具有良好的线性关系,符合碳数变化规律。线性回归方程的斜率可以反映固定相和溶质之间疏水相互作用的强度,CBS柱的斜率(0.13)小于C_18_柱(0.18),这表明CBS柱的疏水性弱于C_18_柱。这主要是由于腰果酚的键合量较低,其烷基链较短,并且在开环反应中引入了极性官能团羟基。

**图5:**
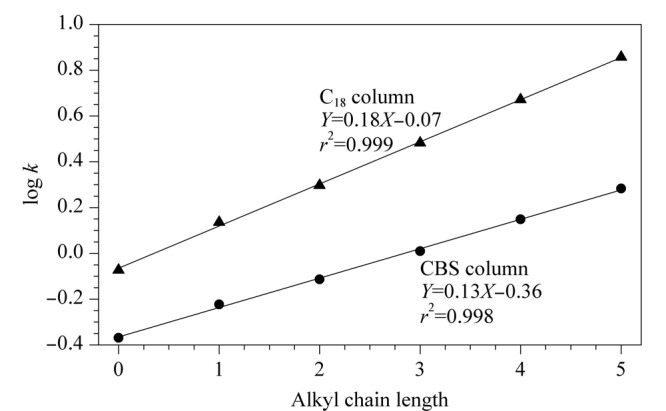
CBS柱和C_18_柱上烷基苯侧链长度与log *k*关系

### 2.3 CBS柱的色谱性能考察

#### 2.3.1 Tanaka实验

Tanaka实验常用于评估反相色谱的疏水性、形状选择性(区分平面和非平面溶质的能力)和硅羟基活性^[21,22]^。实验对比结果如图6所示,分离色谱图如图7所示,戊苯在色谱柱上的保留因子(*k_n_*_-pentylbenzene_, *k*_PB_)取决于固定相中碳含量或配体键合密度,CBS柱的*k*_PB_值(3.43)小于C_18_柱(13.18),表明CBS固定相的表面碳覆盖率少于C_18_。戊基苯与丁基苯的保留因子之比( $\alpha_{CH_{2}}$ =*k_n_*_-pentylbenzene_/*k_n_*_-butylbenzene_),称为疏水选择性,通常用于评估色谱柱分离相差一个亚甲基的烷基苯的能力。CBS柱的 $\alpha_{CH_{2}}$ 值(1.42)小于C_18_柱(1.63),这可能是腰果酚键合量较低所致。通过苯并菲与邻三联苯保留因子的比值(*α*_T/O_=*k*_triphenylene_/*k_o_*_-terphenyl_)来评价色谱柱的形状选择性,CBS柱的*α*_T/O_值(2.99)大于C_18_柱(1.44),这表明CBS柱对相对分子质量和大小相似的平面型与非平面型的分析物具有较好的分离性能^[23]^。这也为我们的应用提供了一个新的思路,即可将其用于分析分离具有不同空间构型的化合物。通过咖啡因和苯酚保留因子的比值来评价固定相的氢键作用(*α*_C/P_=*k*_caffeine_/*k*_phenol_)。CBS柱的*α*_C/P_值(1.00)高于C_18_柱(0.52),表明固定相对咖啡因具有更强的保留能力,这是由于KH-560偶联剂开环反应时形成的羟基能与咖啡因之间形成氢键作用。戊基苯和邻三联苯的保留因子之比用来评价固定相对芳香族化合物的选择性(*α*_PB/O_=*k_n_*_-pentylbenzene_/*k_o_*_-terphenyl_)^[24]^, C_18_柱的*α*_PB/O_值(1.12)大于CBS柱(0.57),且C_18_柱的*α*_PB/O_值大于1,表明在C_18_柱上邻三联苯在戊基苯之前被洗脱出来。相反CBS柱的*α*_PB/O_<1,表明具有更多芳环结构的邻三联苯在CBS柱上的保留能力更强,这主要是由于腰果酚中所带的苯环与溶质中的苯环结构具有*π-π*相互作用,从而增强了色谱柱对溶质的保留能力^[25]^。利用苯胺和苯酚的保留因子之比(*α*_B/P_=*k*_benzenamine_/*k*_phenol_),来评估固定相表面硅羟基活性。尽管CBS固定相的表面键合量较低,并且引入了羟基,但CBS柱的*α*_B/P_值(0.54)小于C_18_柱(1.32)。这主要是由于CBS固定相除疏水作用外还具有氢键作用和*π-π*作用,从而增强了固定相对苯酚的保留作用,此外配体的空间位阻也会对硅羟基活性起到屏蔽作用^[24,26]^,因此CBS固定相的*α*_B/P_值较小。

**图6:**
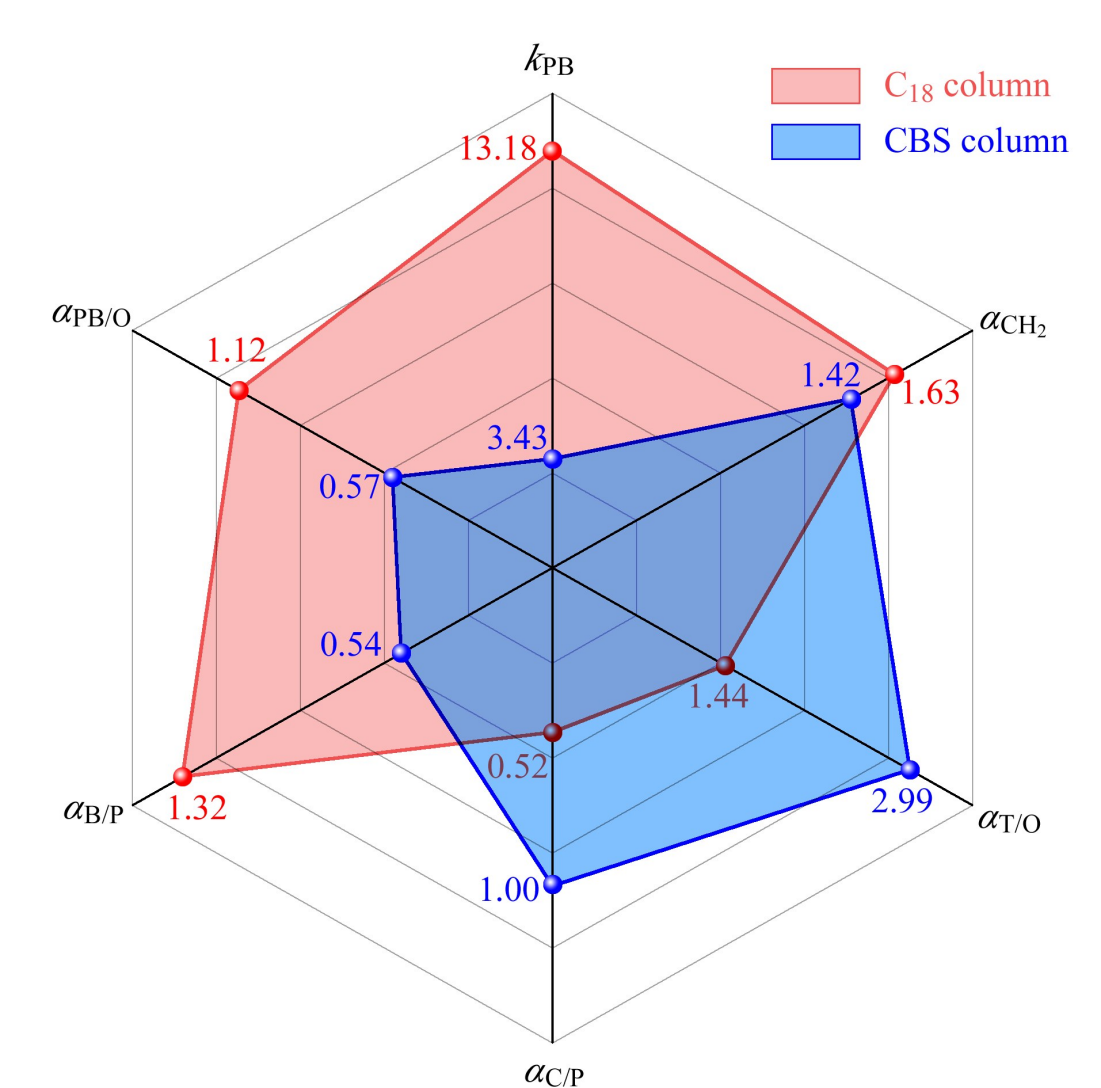
CBS柱与C_18_柱的Tanaka参数雷达图

**图7:**
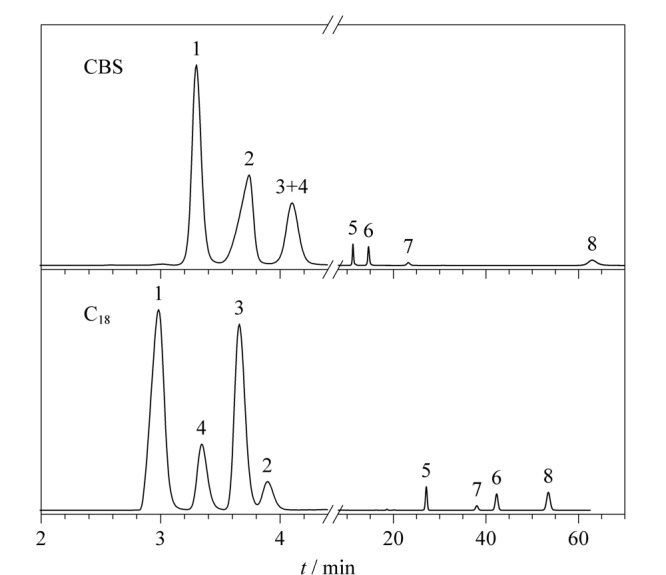
CBS柱与C_18_柱的Tanaka色谱图

#### 2.3.2 分离多环芳烃

多环芳烃作为日常生活中常见的有机污染物,对其进行准确分离和测定至关重要。为考察CBS柱对多环芳烃的分离性能,选用了7种多环芳烃测试其分离性能,并与C_18_柱对比。分离结果如图8所示,多环芳烃的洗脱顺序与其疏水性一致,色谱峰形良好。值得注意的是,尽管CBS柱的疏水作用弱于C_18_柱,但在相同的色谱条件下,CBS柱对多环芳烃的保留性能与C_18_柱相差较小,且芘和苯并[*a*]蒽在CBS柱上的保留时间大于C_18_柱。这主要是由于芘和苯并[*a*]蒽中存在较多的苯环结构,能与固定相上的苯环形成更强的*π-π*作用,从而使CBS柱对其具有更强的保留能力。

**图8:**
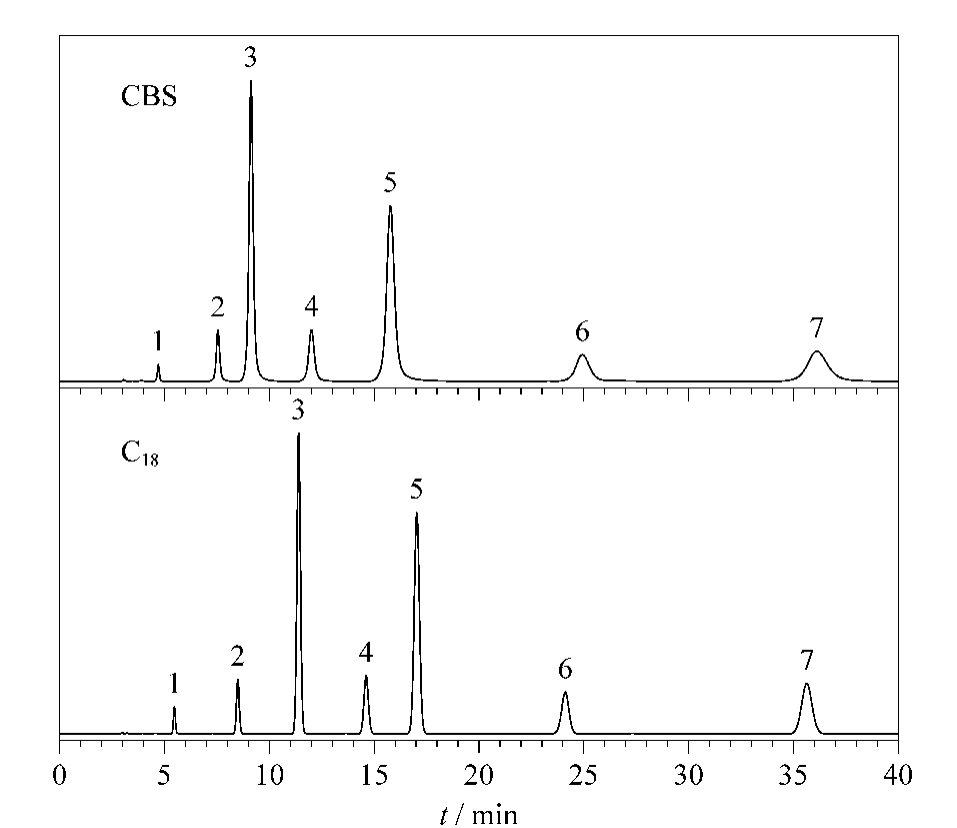
多环芳烃在CBS和C_18_柱的分离色谱图

为进一步研究芳香选择性对烷基苯和多环芳烃保留性能的影响,以烷基苯和多环芳烃保留因子的对数log *k*与其油水分配系数的对数 log *P*进行线性拟合,对其芳香选择性进行评价^[27,28]^。结果如图9所示,在C_18_上拟合得到的两条直线斜率相差较小且几乎相同,且烷基苯直线位于多环芳烃直线的上方,表明这两类溶质在C_18_柱上保留机制仅与其疏水作用有关。但在CBS柱上拟合得到的两条直线斜率不仅相差较大,并且烷基苯直线位于多环芳烃直线的下方,说明CBS柱对多环芳烃具有更强的保留能力。例如,萘的log *P*值小于丙苯,但其在CBS柱上的log *k*值却大于丙苯,表明多环芳烃在CBS柱上的保留性能是疏水和*π-π*作用协同作用的结果。

**图9:**
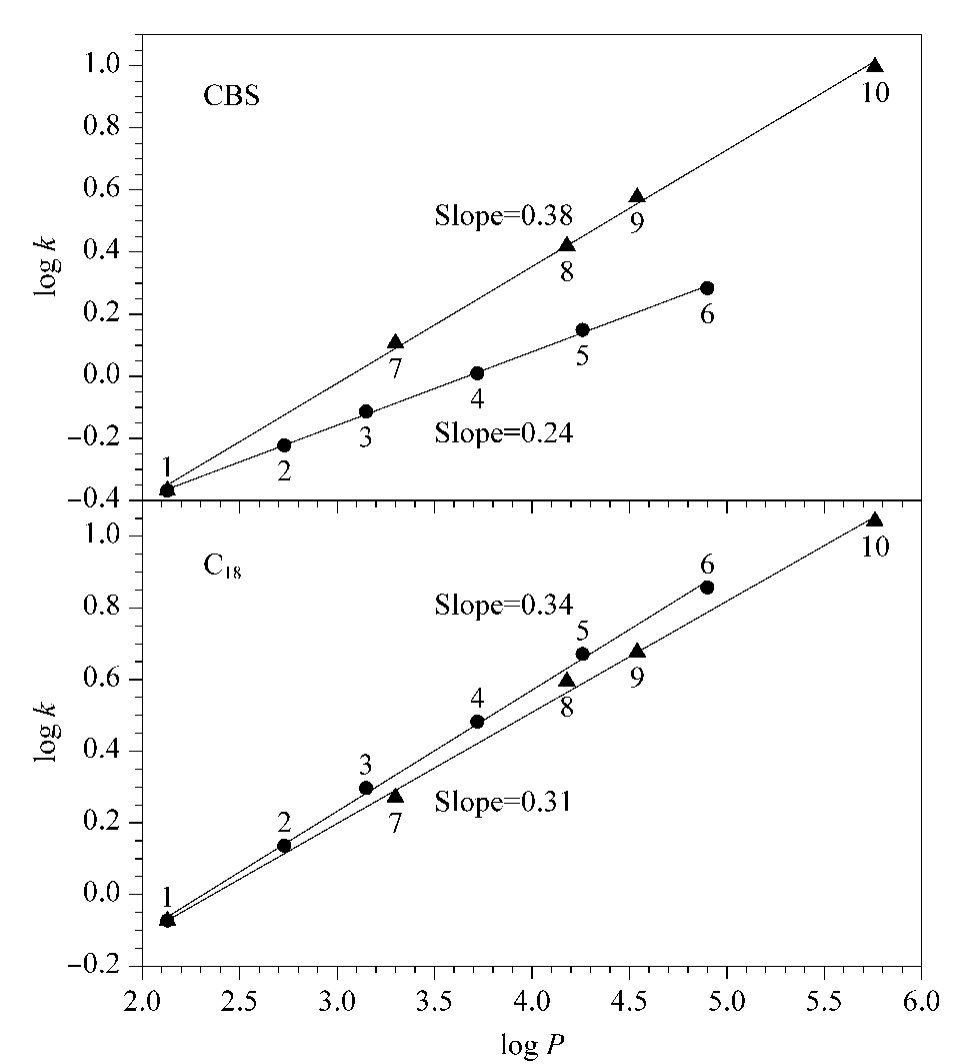
溶质在CBS和C_18_柱上log *k*与log *P*之间的关系

此外,另一类多环芳烃邻三联苯、间三联苯和苯并菲也被用于评价CBS柱的保留性能,并与C_18_柱进行对比。从图10可以看出在相同色谱条件下,3种多环芳烃在CBS柱上成功实现了基线分离,但在C_18_柱上间三联苯和苯并菲的色谱峰完全重叠在一起。与非共平面构型的间三联苯相比,共平面构型的苯并菲能够形成更强的*π-π*作用,从而增强了CBS柱对邻三联苯、间三联苯和苯并菲的分离选择性,这也与Tanaka实验中*α*_T/O_的评价结果相符。

**图10:**
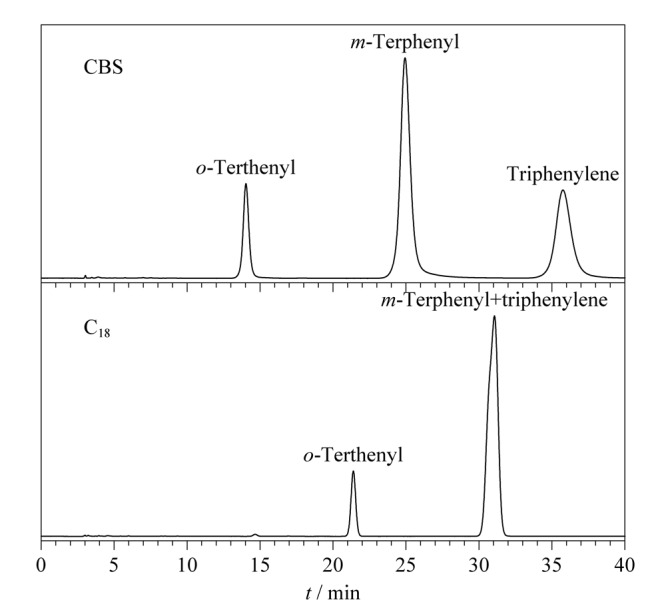
多环芳烃异构体在CBS柱和C_18_柱上的色谱图

#### 2.3.3 分离酚类化合物

CBS固定相表面存在的大量-OH基团是良好的氢键作用位点。以间苯三酚、间苯二酚、苯酚、2-甲酚、3-硝基苯酚和2-萘酚为探针研究了氢键作用对CBS柱分离性能的影响。由图11可以观察到,CBS柱和C_18_柱对6种酚类化合物均表现出良好的分离性能,但酚类化合物在两根色谱柱上的保留性能存在明显的差异,除了2-甲酚和3-硝基苯酚,各探针的出峰顺序与物质的疏水性一致。CBS柱对3-硝基苯酚表现出更强的保留性能,除了*π-π*作用外,固定相表面的-OH基团和3-硝基苯酚的-NO_2_基团形成氢键相互作用。尽管C_18_柱的疏水作用强于CBS柱,但CBS柱对间三苯酚、间苯二酚和2-萘酚的保留性能均强于C_18_柱,表明*π-π*作用和氢键作用在一定程度上可以弥补CBS柱疏水作用较弱的缺陷,另一方面也表明CBS柱对苯酚类化合物的成功分离是疏水、*π-π*和氢键作用协同作用的结果。

**图11:**
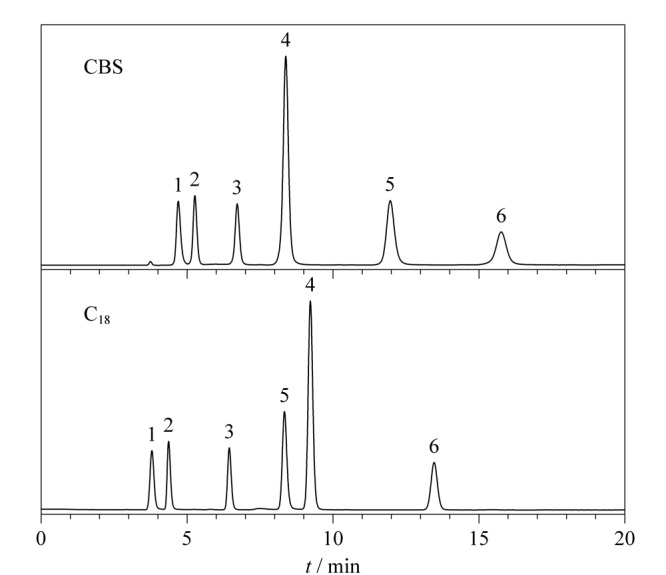
苯酚类化合物在CBS柱和C_18_柱上的色谱图

#### 2.3.4 色谱柱稳定性考察

在应用过程中,色谱柱需要具有良好的稳定性。为了评价色谱柱的稳定性,以烷基苯为探针重复进样35次,随机选取8次分离色谱图,叠加后如图12所示,其色谱峰所对应的物质分别为苯、甲苯、乙苯、丙苯、丁苯、戊苯,由图12可知各探针的色谱峰基本重叠。选取前10次分离结果,对各烷基苯的保留时间和峰面积进行统计,其中各烷基苯保留时间的RSD≤0.079%,色谱峰峰面积的RSD≤0.847%,色谱峰峰高的RSD≤0.272%。各烷基苯的保留时间、峰面积和峰高几乎未发生改变。此外,重新制备了2批CBS固定相,并将其填充到柱管,3批色谱柱对6种酚类物质保留时间的RSD为1.41%~9.26%,表明CBS柱具有良好的稳定性和重复性。

**图12:**
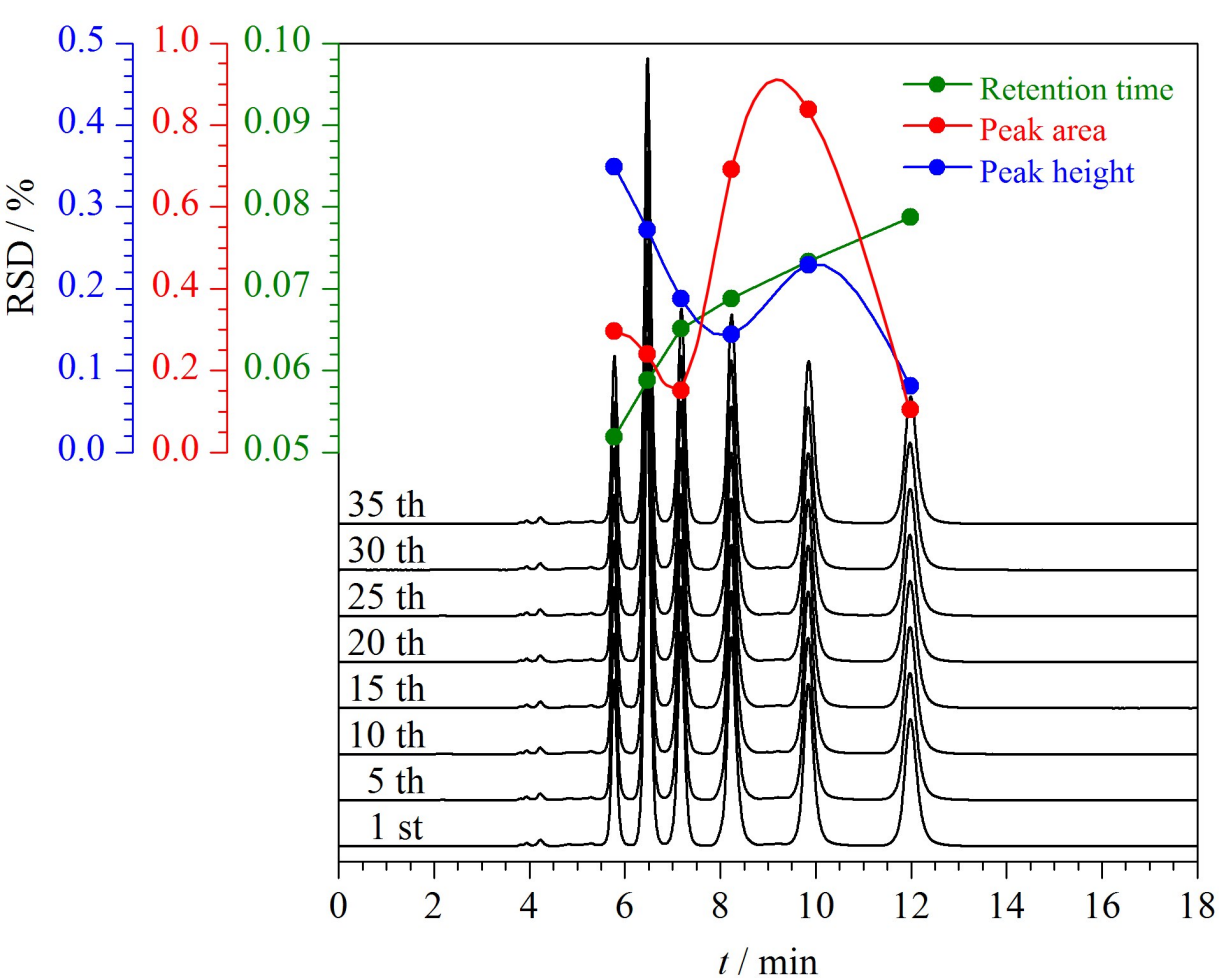
CBS柱的分离重复性

### 2.4 CBS柱分离喜树果和吴茱萸粗提物

以乙腈/水(40∶60, v/v)为流动相,柱温25 ℃,进样量10 μL,检测波长254 nm,流速为1.0 mL/min,测试CBS柱对喜树果粗提物的分离性能,分离结果如图13a所示。CBS柱对喜树果粗提物展现出良好的分离性能,其中峰形最高的色谱峰与喜树碱对照品色谱峰保留时间一致,且峰形良好。喜树碱与前后相邻的两个色谱峰的分离度分别为4.23和2.71。

**图13:**
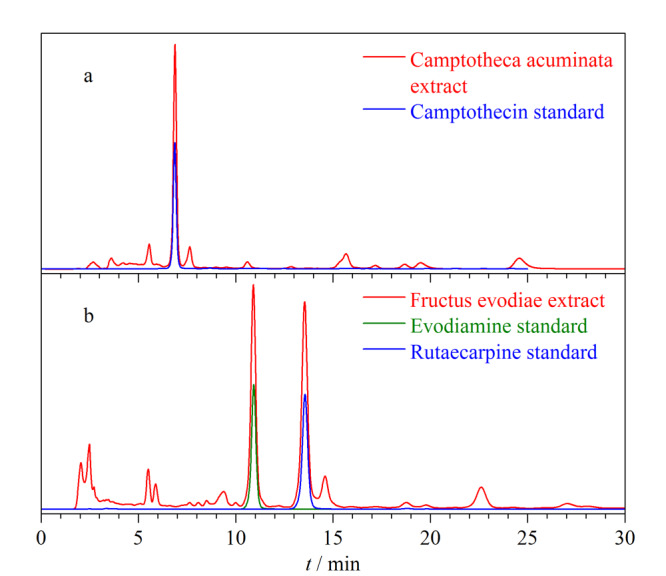
(a)喜树果粗提物和(b)吴茱萸粗提物在CBS柱上的色谱图

以乙腈/水(60∶40, v/v)为流动相,柱温25 ℃,进样量10 μL,检测波长290 nm,流速为1.0 mL/min,测试CBS柱对吴茱萸粗提物的分离性能,分离结果如图13b所示,其中峰形最高的两个色谱峰分别与吴茱萸碱和吴茱萸次碱对照品色谱峰保留时间一致,且峰形良好。吴茱萸碱与吴茱萸次碱的分离度为5.43,与前后相邻的两个色谱峰的分离度分别为2.20和1.69。上述结果表明,CBS柱对这两味中药的主要活性成分分离具有一定的潜能,在后续工作中会对其他的色谱峰进行进一步的结构鉴定。

## 3 结论

本文以绿色可再生资源腰果酚为配体,制备得到腰果酚键合硅胶色谱固定相并对其色谱性能进行了考察。制备出的腰果酚键合硅胶色谱具有典型的反相色谱保留机制,通过Tanaka实验对其色谱性能进行评价,发现该色谱柱除疏水作用外还具有*π-π*作用和氢键作用,并对烷基苯、多环芳烃和苯酚类化合物表现出良好的分离选择性。腰果酚键合硅胶色谱柱对中药喜树果和吴茱萸的粗提物表现出良好的分离性能,为喜树碱和吴茱萸碱的分离纯化提供了新的选择;该固定相制备方法简单,配体资源丰富,有望用作工业色谱分离材料,用于部分中药的分离纯化。
